# Disease control in patients with asthma and respiratory symptoms (wheezing, cough) during sleep

**DOI:** 10.1186/s40733-020-00062-w

**Published:** 2020-09-23

**Authors:** Jonathan Doenges, Elisabeth Kuckuck, Werner Cassel, Olaf Hildebrandt, Andreas Weissflog, Keywan Sohrabi, Niklas Koehler, Volker Gross, Timm Greulich, Ulrich Koehler

**Affiliations:** 1 Department of Internal Medicine, Division of Pneumology, Intensive Care and Sleep Medicine, Hospital of the University of Marburg, Baldingerstrasse 1, 35033 Marburg, Germany; 2 Thora Tech GmbH, Gießen, Germany; 3 University of Applied Sciences, Faculty of Health Sciences, Gießen, Germany

**Keywords:** Asthma, Wheezing, Cough, Long-term monitoring of respiratory sounds, Asthma control test

## Abstract

**Introduction:**

The *Global Initiative for Asthma (*GINA)-defined criteria for asthma control include questions about daytime symptoms, limitation of activity, nocturnal symptoms, need for reliever treatment and patients’ satisfaction. Patients with nocturnal symptoms like wheezing and cough often suffer from lower sleep quality and impaired daytime performance. The lack of an appropriate method for standardized and objective monitoring of respiratory symptoms leads to difficulties in asthma management. The aim of this study is to present a new method for automated wheeze and cough detection during sleep and to assess the actual level of asthma control by the Asthma Control Test (ACT).

**Methods:**

Respiratory symptoms like wheezing and cough were recorded with the LEOSound-Monitor for one night in 55 asthmatic patients in their individual domestic setting. Patients were asked to assess their level of asthma subjectively with the ACT. The study consisted of 37 women and 18 men, with a mean age of 41 years, and a mean BMI of 27 kg/m^2^. Most of the patients had been taking an ICS/LABA combination and would resort to a SABA as their rescue medication.

**Results:**

60% of the participants were classed as having controlled, and 40% were classed as having partially- or uncontrolled asthma. During sleep wheezing was found in 8 of the 55 asthma patients (14.5%) and coughing was found in 30 patients (54.5%). The median ACT score in wheezing-patients was 14, while in non-wheezing patients it was 21. Uncontrolled asthma was found in 6 of the 8 wheezing-patients. Coughing versus non-coughing patients did not show a significant difference in the ACT-score (20, 22 respectively).

**Conclusion:**

Wheezing is a sign of uncontrolled asthma. The ACT-score in wheezing patients is worse compared to patients without wheezing. LEOSound proofed to be a useful tool in providing an objective evaluation of respiratory symptoms, like coughing and wheezing. In clinical practice, this may allow an improvement in asthma therapy.

## Introduction

Bronchial asthma is one of the most common chronic diseases affecting worldwide about 300 Million people of all ages and of all ethnic backgrounds. Furthermore, a lack of optimal medical care is described as a factor of preventable deaths [1]. Medication management of asthma is based on the degree of asthma control. GINA-defined criteria for asthma control include questions about daytime symptoms, limitation of activity, nocturnal symptoms, need for reliever treatment and patient satisfaction [2]. Effective asthma control is necessary to prevent exacerbations and worsening of lung function. Standardised and validated questionnaires such as the Asthma Control Test (ACT) assess the level of asthma control [3]. It classifies asthma as “controlled”, “partially controlled” or “uncontrolled” within the last 4 weeks.

Multicentre studies like REALISE and AIRE provide information about effectiveness and adherence to medication over nearly 15 years. Asthma is still poorly controlled in more than 50% of patients despite the availability of very effective drugs [4–10]. In addition, acoustic long-term monitoring of respiratory sounds is an important addition to the diagnostic spectrum, because symptoms like wheezing and cough are objectively measurable, especially during sleep [11, 12]. The patient’s perception of disease and the reality diverge considerably sometimes [13]. A large proportion of asthma patients rate their disease control as good when in fact it is not. Therefore, an objective evaluation of the respiratory symptoms in patients with asthma may have a huge clinical impact by classifying the asthma control status more accurately. Consequently, medication and therapy of patients with asthma could be improved.

The aim of this study was i.). Analyse the patients’ judgement of asthma control and ii.). Monitor respiratory symptoms like wheezing and cough during sleep.

## Methods

We subselected 55 patients from a previous, not yest published, study, which focused on the phenotypes of eosinophilic and non-eosinophilic asthma. Inclusion criteria were an age between 18 and 65 years as well as a diagnosis of bronchial asthma. In addition, we limited the patients’ nicotine abuse to a maximum of 10 pack years. Informed written consent to participate in the study was obtained from every patient. Exclusion criteria were the presence of any acute lung disease (e.g. bronchitis) or severe infectious disease (e.g. tuberculosis, etc.). Before the overnight study took place, the measurement procedure was explained to the patient, including the positioning of the three microphones. The microphones and the device were designed to ensure maximum comfort, which resulted in a high compliance of the participants. The duration of the overnight monitoring was between 7 and 9 h. The data collection took place between September 2018 and October 2019. Besides, the patients were asked to assess their level of asthma with the ACT. The study was approved by the Ethics Committee of the Philipps University of Marburg (Az.: 94/18).

### Longterm recording of respiratory sounds - LEOSound

**Fig. 1:**
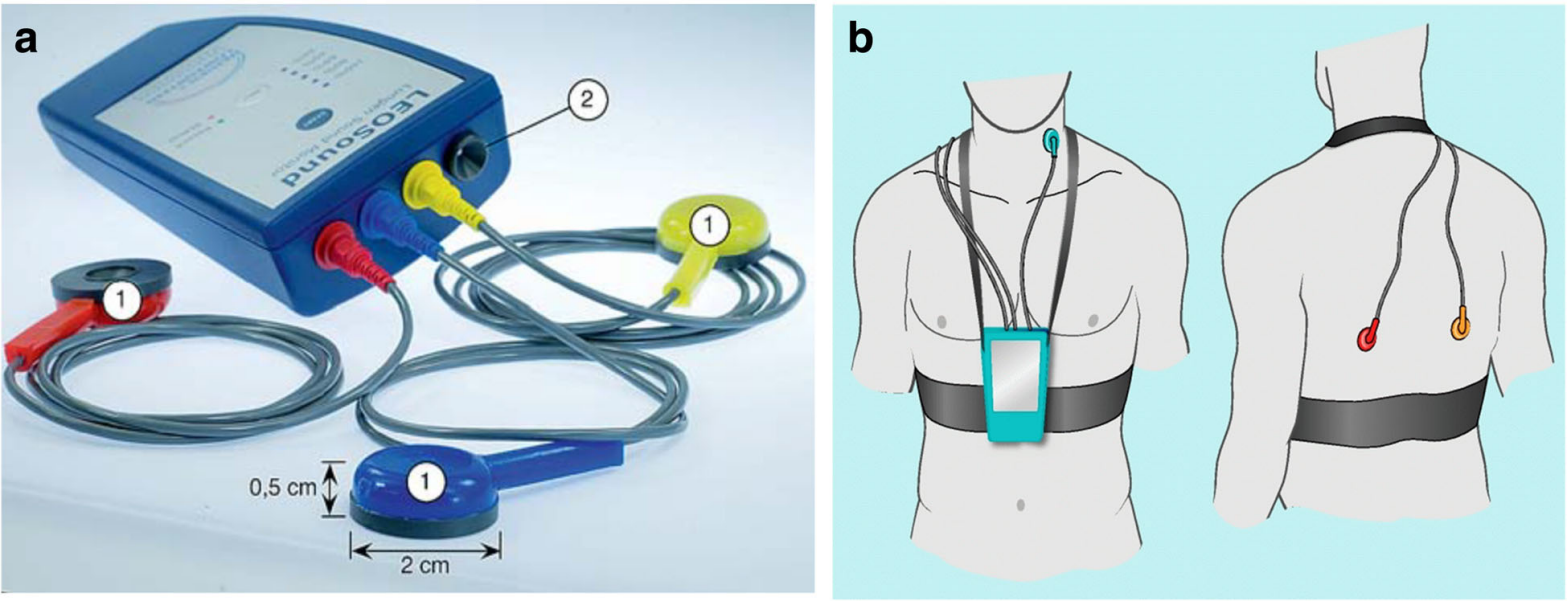
**a** LEOSound-recorder with three body microphones (labelled 1) and one ambient microphone (labelled 2). **b** Illustration of the recorder and microphone placement during the observation. The figure depicts one tracheal microphone and two bronchial microphones [11]

**Fig. 2:**
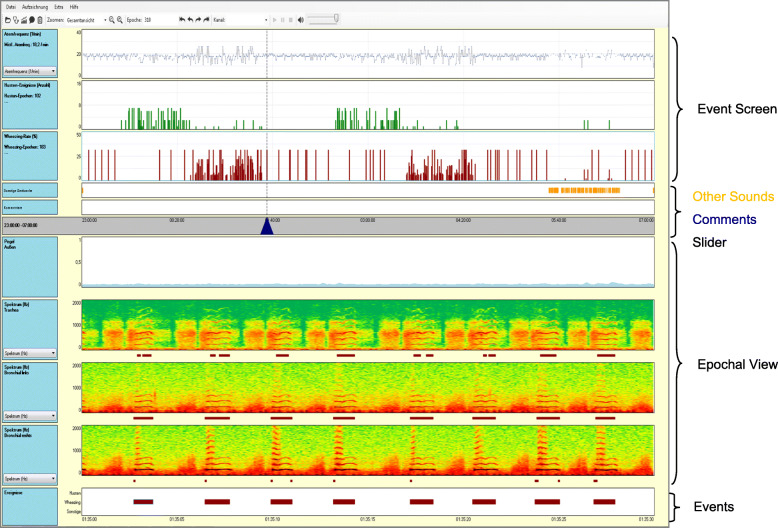
The user interface of the LEOSound-Analyzer is divided into two major fields - shown as **Event Screen** and **Epochal View**. The **Event Screen** displays the breathing rate (blue line), the detected coughing events (green) and the wheezing rate (red) of the associated epoch. In the **Epochal View** the selected epochs from the Event Screen are displayed as a spectrogram. Optionally, the epochs can be represented as a loudness level

The LEOSound Lung-Sound-Monitor is a mobile device validated for automatic long-term recording and analysis of normal and adventitious respiratory sounds like cough and wheezing in adults and children [11]. The system automatically detects cough and wheezing for up to 24 h and can be used either in the hospital or at the patient’s home. Sound is recorded with three bio-acoustical sensors, one placed at the trachea and two placed on the patients’ back (Fig. 1). In addition, an ambient microphone is integrated in the LEOSound device. It is thus possible to differentiate lung sounds from speech and other ambient sounds. The devices were programmed in advance for every patient. The associated software contains automated algorithms for cough and wheezing detection (Fig. 2). The LEOSound-Monitor was validated in various clinical studies, and showed a sensitivity and specifity of 80–95% [14].

### Patients

Thirty-seven women and 18 men, aged between 20 and 67 years, were included. The average age of the participants was 41 years with a mean BMI of 26.5 kg/m^2^ (standard deviation ±6.1). Table 1 gives an overview of the patients’ characteristics. There was no restriction in medication. Most of the included patients had been taking an ICS/LABA combination and would have resorted to a SABA as their rescue medication (see Table 2).

**Table 1:** Anthropometric data of study cohort (*n* = 55)

	n/N or Average ± SD	Min - Max
Sex [% female]	37/55 (67.3)	
Age [years]	41.0 ± 13.7	20–67
Weight [kg]	78.9 ± 21.2	51–136
Height [cm]	172 ± 12	154–200
BMI [kg/m^2^]	26.5 ± 6.1	17.4–47.1
Smoker [PY]	1.8 ± 3.2	0–10

**Table 2:** Medication used within the study cohort

Medication	n/N (%)
ICS/LABA	37/55 (67,3)
ICS solo	7/55 (12,7)
LAMA	8/55 (14,5)
SABA	39/55 (70,9)
SAMA	4/55 (7,3)
Omalizumab	3/55 (5,5)
Montelukast	7/55 (12,7)
Mepolizumab/Benralizumab	3/55 (5,5)

### Asthma control test (ACT)

Asthma control was determined using a validated questionnaire (Asthma Control Test, ACT) [3]. We decided to choose the ACT cut of scores that GINA referred to in its main report [2]. This resulted in a more balanced data set. The scoring system reflects the degree of asthma control during the previous month, and is based on five questions: 1) shortness of breath, 2) awakenings due to asthma symptoms, 3) frequency of reliever medication use, 4) impairment at work or school and 5) patient’s own rating of control. The patient rates each criterion with a score from 1 (worst) to 5 (best). 20–25 points represent an asthma well under control (ACT-1), a score of 16–19 points indicates a partially controlled asthma (ACT-2), whereas 15 or less points indicate an uncontrolled asthma (ACT-3).

### Statistical methods

Statistical analysis was performed using IBM SPSS Statistics Version 25 (IBM GmbH). The Kolmogorov-Smirnov-Test was used to test for normal distribution. Where the normal distribution hypothesis was rejected, non parametric methods were used for both descriptive and interferential statistical calculations. As coughing and wheezing variables were not found to be normally distributed, median and range were used to describe all parameters. For some normally distributed variables such as age, average and standard deviation (AVG ± SD) were reported as well.

Kruskall-Wallis one-way analysis of variance was used to compare the three Asthma Control groups. A statistically significant test result was obtained, if *p* ≤ 0.05.

The Mann-Whitney test was also used to compare wheezing and non-wheezing patients and coughing and non-coughing patients.

## Results

60% of the patients were classed as having controlled asthma, and 40% of the patients as having partially- or uncontrolled asthma. In 33 patients the asthma status was classified as ACT-1, in 10 patients as ACT-2 and in 12 patients as ACT-3. Table 3 shows the patients’ parameters depending on their asthma control status.

**Table 3:** Differences between anthropometric data, and parameters of LEOSound-monitoring depending on ACT status

	ACT – 1 (*n* = 33)		ACT – 2 (*n* = 10)		ACT – 3 (*n* = 12)		
	Median	Min – Max	Median	Min – Max	Median	Min - Max	*p*-value (over all/ACT-1 vs 3)
Collective of patients							
Age	34	20–63	38	23–67	55.5	30–67	**0.003/0.001**
Height	172.5	154–200	167	159–195	163.5	156–196	0.217
Weight	76.5	52–131	70	51–136	78.5	55–135	0.752
BMI	24.8	17.4–36.7	23.7	18.7–47.1	26.7	21.8–40.8	0.443
PY	0	0–10	0	0–10	1	0–8	0.389
ACT	22	21–25	18	16–20	14	10–15	**< 0.001/< 0.001**
LEOSound							
	Median	Min – Max	Median	Min - Max	Median	Min - Max	*p*-value
Cough epoch /hour	0	0–0.8	0.1	0–1.8	0.6	0–4.8	**0.040/0.015**
Abs Quantity coughs	0	0–5	3	0–28	10	0–275	0.051
Quantity of cough /h	0	0–25	0.4	0–3.5	1.3	0–34.4	**0.049/0.017**
Wheezing phases	0	0–1	0	0–3	0.5	0–5	**< 0.001/< 0.001**
Wheez. epoch/hour	0	0–1	0	0–7.6	0.8	0–7.3	**< 0.001/< 0.001**
Wheez. durat. [min]	0	0–8.9	0	0–30.5	3	0–29	**< 0.001/< 0.001**
Wheez. duration [%]	0	0–0.9	0	0–8.1	0.8	0–6.5	**< 0.001/< 0.001**

Statistically significant *p*-values are bold

### Wheezing

**Fig. 3:**
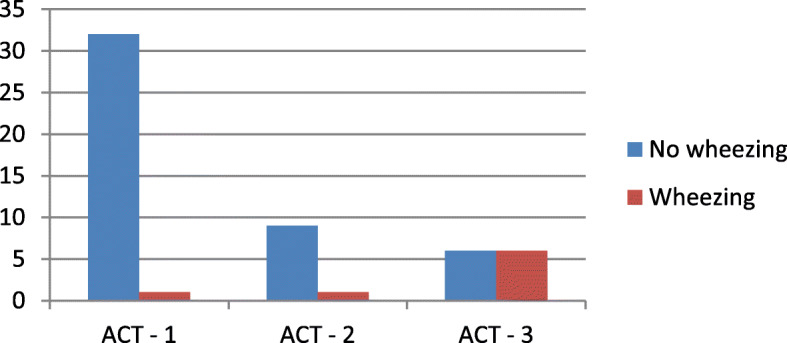
Nocturnal wheezing and ACT-grouped patients

Lung sound recordings showed wheezing during sleep in 8 out of 55 patients (14.5%) with a median duration of 9.5 min. The maximum wheezing duration was 30.5 min. With respect to ACT, the median score of the non-wheezing group was 21, whereas within the wheezing group the median score was 14 points. Six out of 8 wheezing patients were found in the ACT-3 Group, which means their current asthmatic status was uncontrolled. Statistical calculation showed a significant difference between the wheezing- and the non-wheezing group (*p* = 0.001). Table 4 compares the anthropometric data and the recorded LeoSound parameters between the wheezing and the non- wheezing group. Figure 3 shows the total number of wheezing patients across the different ACT-groups.

**Table 4:** Differences between anthropometric data, ACT scores and LEOSound-monitoring in patients with and without wheezing

	No wheezing (*n* = 47)		Wheezing (*n* = 8)		
	Median	Min – Max	Median	Min – Max	*p*-value
Collective of patients					
Age [years]	37	20–67	54	38–67	**0.01**
Height [cm]	171	154–200	164	156–182	0.25
Weight [kg]	76	51–136	75	55–135	0.94
BMI [kg/m^2^]	24.9	17.4–47.1	25.1	22.0–40.8	0.38
Pack years	0	0–10	0,5	0–8	0.58
ACT	21	11–25	14	10–22	**0.001**
LEOSound-Monitor					
	Median	Min. – Max.	Median	Min. – Max.	
Cough-Epochs per hour	0.1	0–1.8	0.3	0–4.8	0.25
Abs. quantity of coughs	1	0–63	3	0–175	0.33
Quantity of coughs p. hour	0.1	0–7.9	0.4	0–34.4	0.33
Wheezing-Phases	0	0–0	2.0	1.0–5.0	–
Wheezing-Epochs per hour	0	0–0	2.4	1.0–7.4	–
Wheezing duration [min]	0	0–0	9.5	4.0–30.5	–
Wheezing duration [%]	0	0–0	2.4	0.9–8.1	–

Statistically significant *p*-values are bold

### Cough

**Fig. 4:**
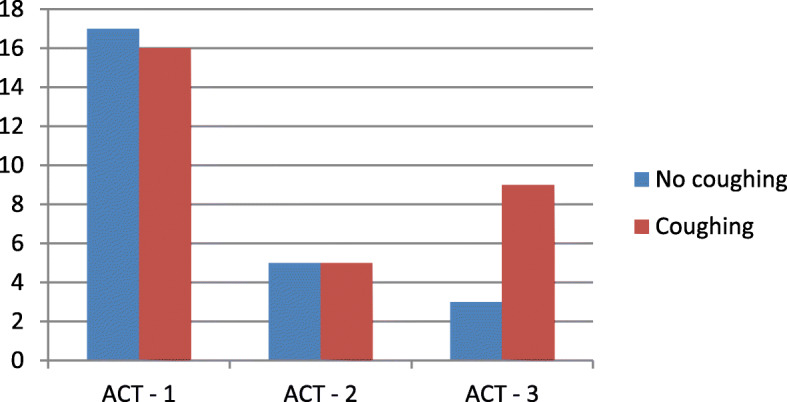
Coughing and non-coughing patients in ACT-groups

Coughing was detected during sleep in 30 patients with a median rate of 0.9 coughs per hour and a maximum of 34.3 coughs per hour. There was no significant difference between the ACT scores of coughing and non-coughing patients. However, as seen in Fig. 4, the ACT-3 Group presented a higher percentage of coughing patients compared to ACT-1 and ACT-2 Group. Table 5 compares the anthropometric data and the recorded LeoSound parameters between the coughing and the non- coughing patients.

**Table 5:** Summary of anthropometric data, ACT scores and LEOSound-monitoring in patients with and without coughing

	No coughing (*n* = 25)		Coughing (*n* = 30)		
	Median	Min – Max	Median	Min – Max	*p*-value
Collective of patients					
Age	33	23–63	46	20–67	0.06
Height	170	154–200	172	156–196	0.85
Weight	78	54–136	76	51–131	0.83
BMI	25.7	19.1–47.1	24.5	17.4–40.2	0.77
Pack years	0	0–10	0.5	0–10	0.38
ACT	22	14–25	20	10–25	0.06
LEOSound-Monitor					
	Median	Min. – Max.	Median	Min. – Max.	
Cough-epochs per hour	0	0–0	0.4	0.1–4.8	–
Absolute quantity of coughs	0	0–0	7.0	1–275	–
Quantity of coughs per hour	0	0–0	0.9	0.1–34.4	–
Wheezing-Phases	0	0–3	0	0–5	0.59
Wheezing-Epochs per h	0	0–7.6	0	0–7.3	0.66
Wheezing duration [min]	0	0–30.5	0	0–29.0	0.66
Wheezing duration [%]	0	0–8.1	0	0–6.5	0.68

## Discussion

In this study, the monitoring of wheezing during sleep showed a significant relation to the ACT score and therefore, the subjective asthma control of patients at daytime. Patients who scored their asthma as being uncontrolled were more likely to show nocturnal wheezing symptoms compared to patients who scored their asthma as controlled. Moreover, the lung sound monitoring during sleep may even detect respiratory symptoms of which patients are unaware, as wheezing was found in one patient who scored his asthma as controlled (see Fig. 3).

Asthma is caused by a multitude of factors and has a heterogeneous set of manifestations [2]. The presentation of asthma is highly variable. Typical asthma symptoms include dyspnoea, coughing, wheezing, occasional thoracic tightness, and severe asthma attacks. Asthma patients frequently suffer from nocturnal and early morning respiratory discomfort, which is due to the chronobiological rhythm of the airway width.

Braghiroli et al. recently described the lack of research and established clinical 24 h monitoring of respiratory symptoms that would result in a more adequate medication therapy. Circadian variations usually cause a worsening of respiratory symptoms during sleep. Supine position and physiological changes such as vagal stimulation with an increased airway resistance are contributing factors. For this reason, Braghiroli et al. emphasized the significance and importance of new digital tools to provide objective and long-term evaluation of respiratory symptoms like cough and wheezing in order to improve therapeutic choice [15].

Wheezes are high-pitched continuous adventitious sounds caused by airway narrowing. Wheezing is a sign of an uncontrolled level of asthma. Automatic detection and classification of cough and wheezing is useful in assisting physicians in the diagnosis and monitoring of acute and chronic respiratory diseases such as asthma, acute bronchitis and COPD [11, 12, 16–18]. Grading of asthma control necessitates specifying the rate of daytime and nocturnal symptoms. This part of classification concept is used nationally and internationally. However, nocturnal symptoms are difficult to evaluate since patients are unaware of them during sleep. Considering asthma therapy and medication, large multicentre studies spanning multiple countries and years provide valuable information about 15 years of asthma control and care on the ground [4–10]. The results are far from satisfactory. One reason may be the lack of objective evaluation of respiratory symptoms during sleep.

Asthma patients often believe that they have good control of their symptoms. However, control of nocturnal symptoms is overestimated. Up to now there is no way of ensuring an objective symptom control. Ding et al. investigated 1115 patients with mild asthma (average age of 38.4 years) with respect to their asthma control [10]. Nearly half of the patients (40.6%) exhibited nocturnal symptoms. However, clearly audible wheezing is rare and constitutes the tip of the iceberg. With acoustic long-term recording of breathing sounds, the wheezing, which is normally only heard through the stethoscope, can be analyzed objectively. In the study by Fletcher and Hiles subjective (parent) and objective (recording) data on the frequency of coughing and wheezing varied massively [13]. Understandably, symptoms occurring during sleep are often not perceived by patients.

In addition, Morice et al. presented in their guidelines the need for an objective assessment of respiratory symptoms, like cough and wheezing [19]. So far, evaluation of respiratory symptoms are basically estimated through subjective descriptors. Procedures like manual counting of coughs and wheezing episodes are used in an attempt to obtain a quantitative and objective component. However, for the evaluation of large numbers of patients this approach is impractical. Consequently, there is a necessity for an automated/ objective evaluation of respiratory symptoms [20].

In clinical practice, cough monitors are already in use. After significant progress and development in objective cough monitoring, tools are ready for standard deployment [21]. Nevertheless, long-term monitoring of wheezing has not yet been established in clinical practice.

In this study we only included adults. However, we expect objective monitoring of respiratory symptoms like cough and wheezing to have an even greater impact on children. Asthmatic children and adolescents are more likely to show impaired daytime performance compared to healthy controls [22–25]. Night-time asthma results in poorer sleep quality that can also have adverse effects on their efficiency during daytime [20, 26]. Reports of children symptoms are often inaccurate or unusable and therefore it becomes essential to objectify respiratory symptoms such as coughing and wheezing, especially during sleep. The acoustic long-term recording of adventitious respiratory sounds is an important addition to the diagnostic spectrum. It is readily available and provides a symptom assessment that is easy to comprehend for, the doctor as well as the patient. Therefore, objective evaluation of respiratory symptoms like cough and wheezing may allow a more accurate and adequate therapy in children with asthma, resulting in a profound effect on children’s health.

International multicentre studies such as AIRE (2000) and REALISE (2014) provide information about the reality of asthma treatment and control [4, 5]. Despite the availability of effective drug therapy, patients with asthma are still insufficiently controlled. By several major studies 50–80% of all patients were classed as having uncontrolled or just partially controlled status. By implication, only a quarter of the patients were considered to have well-controlled asthma.

In 2014 Price et al. published the REALISE study, in which more than 8000 asthma patients from 11 European countries were surveyed for asthma control [5]. In this study 45% of patients showed uncontrolled asthma, while 44% of the patients exhibited at least one acute exacerbation in the previous year, which necessitated oral steroid treatment. 12% of the patients had to be hospitalized. Over 80% of respondents rated their asthma as well controlled! In a previous study (AIRE) published by Rabe et al. 2000, 80% of respondents also rated their own asthma control as good, however, objective scrutiny showed that to be the case in only 20% of those surveyed [4]. While an improvement from 45 to 80% controlled asthma is a step in the right direction, considerably more improvement has yet to be achieved. Accordingly, in this study only 60% of the patients scored their asthma as controlled by using the ACT. Moreover, a rating of controlled asthma did not exclude wheezing symptoms during sleep, indicating a lack of subjective asthma control scoring.

The study by Olaguibel et al. revealed that uncontrolled asthma, as assessed by the Asthma Control Questionnaire (ACQ), may be independent of asthma severity [6]. The study included 1363 stable asthmatics with a mean age of 38 years. Only 13.6% of the patients were considered to have controlled asthma, partially controlled asthma was found in 34.2% of the patients, and 52.3% of the patients were deemed to have uncontrolled asthma.

### Limitations

In this study, patients were monitored for only one night. Although asthma has a large symptom variability, and respiratory symptoms may differ from one night to another. Nevertheless, the monitoring for one night seems relevant since respiratory symptoms were detected. The aim of the study was to highlight the necessity for and the clinical impact of objective monitoring of respiratory symptoms like cough and wheezing in patients with asthma. Moreover, the sample size of only 55 patients is arguably a limitation of this study. A larger sample size may provide more solid evidence. However, analysis and statistical calculation of our data already showed an appropriate application of this study with respect to 55 patients.

## Conclusion

In our opinion, cough and wheezing during sleep have a direct impact on sleep and quality of life in patients with asthma. Consequently, for the assessment and evaluation of wheezing and cough events, objective approaches like respiratory sound monitoring seem most appropriate. This study presented that a device for long-term monitoring of respiratory sounds is a helpful tool to monitor time course of cough and especially wheezing in patients with obstructive respiratory diseases. Generally, patients may not necessarily notice the respiratory symptoms during sleep. Therefore, the objective evaluation may have a huge clinical impact by adjusting and improving the therapy according to the respiratory symptoms. Moreover, this study only focused on adults whereas the objective evaluation of respiratory symptoms seems to have even more profound relevance on children’s health. Especially small children are not as able as older patients to notice and report their respiratory symptoms. This, further research including children may demonstrate. In addition, polysomnographic research may elucidate the impact of sleeping behavior in wheezing and non-wheezing patients on daytime performance and productivity.

## Data Availability

The datasets generated and analysed during the current study are available from the corresponding author on reasonable request.

## Abbreviations

ACT: Asthma control test
AVG: Average
BMI: Body mass index
GINA: Global initiative for asthma
PY: Pack years
ICS: Inhaled corticosteroid
LABA: Long acting beta-sympathomimetic agonist
SABA: Short acting beta-sympathomimetic agonist
LAMA: Long acting muscarinic antagonist
SAMA: Short acting muscarinic antagonist
SD: Standard deviation
